# Ps19, a novel chitin binding protein from *Pteria sterna* capable to mineralize aragonite plates *in vitro*

**DOI:** 10.1371/journal.pone.0230431

**Published:** 2020-03-19

**Authors:** Raquel G. Arroyo-Loranca, Norma Y. Hernandez-Saavedra, Luis Hernandez-Adame, Crisalejandra Rivera-Perez

**Affiliations:** 1 Molecular Genetics Laboratory, Centro de Investigaciones Biológicas del Noroeste (CIBNOR), La Paz, Baja California Sur, México; 2 CONACYT, Centro de Investigaciones Biológicas del Noroeste (CIBNOR), La Paz, Baja California Sur, México; University of California, UNITED STATES

## Abstract

Mollusk shell is composed of two CaCO_3_ polymorphs (calcite and aragonite) and an organic matrix that consists of acetic acid- or ethylenediaminetetraacetic acid (EDTA)-soluble and insoluble proteins and other biomolecules (polysaccharides, β-chitin). However, the shell matrix proteins involved in nacre formation are not fully known. Thus, the aim of this study was to identify and characterize a novel protein from the acetic acid-insoluble fraction from the shell of *Pteria sterna*, named in this study as Ps19, to have a better understanding of the biomineralization process. Ps19 biochemical characterization showed that it is a glycoprotein that exhibits calcium- and chitin-binding capabilities. Additionally, it is capable of inducing aragonite plate crystallization *in vitro*. Ps19 partial peptide sequence showed similarity with other known shell matrix proteins, but it displayed similarity with proteins from *Crassostrea gigas*, *Mizuhopecten yessoensis*, *Biomphalaria glabrata*, *Alpysia californica*, *Lottia gigantea* and *Elysia chlorotica*. The results obtained indicated that Ps19 might play an important role in nacre growth of mollusk shells.

## Introduction

The biological process by which an organism crystalizes minerals is known as biomineralization. Calcium carbonate is a mineral that can be biomineralized into three polymorphs: vaterite, aragonite, and calcite; the last two are commonly found in mollusk shell, the second largest group of metazoans [1,2]. In bivalve shell, calcite forms an outer prismatic layer while aragonite forms flat tablets that assemble in a “brick-wall-like” structure or nacre sheet, forming an inner nacreous layer [2–5]. Nacre has outstanding mechanical properties due to the combination of stiffness, strength, and toughness given by its unique microstructure arrangement [6].

Shell formation in mollusks is controlled by epithelial cells from the mantle, the organ responsible for secreting the required components for calcium carbonate crystal biomineralization, such as calcium, carbonate ions and organic macromolecules (proteins, β-chitin, glycoproteins, polysaccharides) that form the organic shell matrix [7,8]. The molecules from the matrix are located either in the inorganic phase (within the calcium carbonate crystal) or between crystals forming the organic framework [9]. The organic matrix guarantees mineral and macromolecule interactions as it has a main role in crystal nucleation and growth [10].

The mechanism of nacre formation has been studied through extraction and functional studies of various shell matrix proteins (SMPs); nevertheless, nacre biomineralization is a very complex process; thus, soluble and insoluble proteins in ethylenediaminetetraacetic acid (EDTA) or 10% acetic acid have been studied because of their biochemical and crystallization nacre properties [11,12]. Previous studies have reported that peptide sequences from insoluble and soluble matrixes share conserved regions or domains; however, shell matrix protein sequences are generally dominated by Asp, Glu, Ser, Ala, Gly, Pro and Cys residues, which are often concentrated in short or long repetitive domains [5,13–15]. Gly and Asn residues usually create specific domains that have been associated with nucleation [16] and inhibition [2,17] functions in the biomineralization process while Gly/Tyr residues or GYS motif have been associated to polysaccharide-binding abilities [2,16–20]. Conservatively, in the insoluble fraction, a combination of chitin-protein complexes exhibit hydrophobic biochemical properties (enrichment in aliphatic amino acids, such as glycine and alanine) while the soluble shell fraction is found to be polyanionic, and particularly aspartic acid-rich [15].

In bivalves, more than 50 different insoluble proteins have been described; some of them are N14/N16/pearlin family, Pif-like family, UNP-family, MSI60-like family, Fam20c, N25, Prismalin-14 [2,3,11,16,18,21–26]. Insoluble acetic acid proteins, such as Pif, MSI60, proteins containing a carbonic anhydrase domain (CA), proteins with LamG (a Ca^+2^ mediated receptor), chitin-binding-containing proteins, together with A-, D-, G, M- and Q-rich proteins, appear to be analogs of proteins previously described from pearl oysters or edible mussel nacre matrices, which constitutes a remarkable set of deeply conserved nacre proteins [11,27]. A proteomic analysis of the insoluble acetic-acid nacre matrices of fresh water mussels showed different domains according to their protein sequences, such as RLCD- (repetitive low-complexity domain), immunity-related, Pif-, WAP-, M-rich, Q-rich, A-rich or chitin binding [11,12,28].

Nacre proteins containing chitin-binding domain (CBD) includes the blue mussel shell protein (BMSP), pearlin/N16 family, hichin, Pif-like, among other proteins. This domain plays an important role in organic scaffold construction following crystal deposition [2,29,30]. Another domain found in insoluble SMPs is the von Willebrand A (vWA) domain, involved in cell adhesion and often associated with CBD, found in proteins as BMSP, Pif family, and others [27,30]. The calcium binding domain is also of vital importance because these proteins are associated with nucleation of aragonite and calcite and found in proteins, such as N16, N14, MSI60 and Aspein [24,31–33].

Besides the intrinsic characteristics of the protein sequences, some SMPs possess posttranslational modifications, such as phosphorylation, glycosylation, and sulfation that determine their structure and their interactions with other molecules; moreover, enzymatic regulations allow them to determine which calcium carbonate polymorph forms by interacting with the mineral [1,17]. For example, pearlin is the only protein of its family, which by itself binds to calcium, and *in vitro* it had the ability to induce aragonite crystals in the presence of Mg^2+^ while N14 mostly combines with N66 (a carbonic anhydrase) to induce aragonite plates; Prismalin-14 binds to Ca^2+^ and displays inhibitory activity on calcium carbonate crystallization in *in vitro* assays [19]. These interactions suggest that posttranslational modifications are crucial for some SMPs to display their role in shell biomineralization [17].

In recent decades, much attention has been drawn to describing shell matrix protein interactions with calcium ions and other biomolecules, as well as their role in nacre formation to understand their contribution to its unique mechanical and biological properties. Many shell matrix proteins remain unknown despite the continuous efforts to isolate, describe and characterize them all. Some of these isolated proteins have not been completely characterized yet, and their role in biomineralization still remains unknown. In this sense, it is important to pursue the efforts to understand the shell biomineralization process. This research aimed to identify and characterize a novel protein called Ps19, the most abundant protein located in the acetic acid-insoluble fraction of the *Pteria sterna* shell, a local pearl oyster of economic importance, to understand its role in shell biomineralization. Surprisingly, this Ps19 exhibited calcium- and chitin-binding capabilities involved in the biomineralization process. These facts revealed new insights in the shell biomineralization mechanisms that were analyzed by crystallization of aragonite plates in *in vitro* studies.

## Materials and methods

### Biological material

Three shells from adult oysters were provided by Perlas del Cortez S. de R.L. MI. located at Bahia de La Paz, B.C.S. Shells were transported to the Molecular Genetics Laboratory facilities at CIBNOR.

### Shell matrix proteins extraction

The organic matrix of the shell of *Pteria sterna* (20 g of pulverized shell) was extracted by decalcification with cold acetic acid (4 °C, 10% *v/v*) as previously described by Montagnani [2]. After decalcification, the solution was centrifuged at 4,500 ×g for 30 min, and the supernatant, Acetic Soluble Matrix (ASM) was collected; the pellet or Acetic Insoluble Matrix (AIM) was collected in a separate tube. The ASM was dialyzed (12–14 kDa cutoff Spectra/Por^®^ membranes, Repligen, No.132680, California, USA) against distilled water overnight at 4 °C under constant low stirring. Then, water exchange was made with distilled water and dialyzed for another 8 h. Afterward, the dialyzed extract (ASM) was concentrated by cold per-evaporation (4 °C, 36 h). The AIM was rinsed with distilled water and water was removed; both fractions (ASM and AIM) were stored at −20 °C for further analysis.

### Sodium dodecyl sulphate-polyacrylamide gel electrophoresis

Sodium dodecyl sulphate polyacrylamide gel electrophoresis (SDS-PAGE) was performed according to Laemmli [34]. Samples ASM (30 μg of protein) and AIM (64 μg of protein) of the shell from *P*. *sterna* were mixed with sample buffer 4× (0.5 M Tris-HCl pH 6.8, 20% glycerol, 10% SDS, 10% β-mercaptoethanol and 0.05% bromophenol blue) and boiled for 10 min, then loaded into a 16% polyacrylamide gel. Broad range molecular weight standard (Bio-Rad 1610317, California, USA) was loaded into the gel. Electrophoresis was conducted at 90-V at room temperature, using a vertical electrophoresis unit (Bio-Rad Protean II, California, USA). After electrophoresis, the gel was stained with Coomassie Brilliant Blue R250 (CBB) for 2 h, washed out and analyzed for protein, using a gel imager (Chemi Doc XRS, Bio-Rad, California, USA). Also, proteins were stained with silver nitrate [35,36]. The same procedure was followed for purified protein from *P*. *sterna* (Ps19), loading 6.8 μg of protein.

### Protein quantification

Quantification of the most abundant protein present in the AIM (Ps19) was performed by pixel densitometry by separating the protein sample in a 16% SDS-PAGE gel and stained with Coomassie Brilliant Blue (R250). First, a standard curve was made with ovalbumin protein (0.25–8.0 μg·μL^-1^); the image was scanned with a Chemi Doc XRS (Bio-Rad, California, USA). Then, the density of the pixels from each band was calculated using the Image Lab 5.1 software, and the linear equation was obtained. The protein amount of Ps19 from the shell of *P*. *sterna* was calculated through the obtained equations of ovalbumin standard curve (*y* = 5 × 10^−6^ *x* − 0.94).

$$y=5\times 10^{-6}x-0.94$$ (1)

### Protein purification by preparative SDS-PAGE

The AIM of *P*. *sterna* was fractionated on a discontinuous preparative polyacrylamide gel electrophoresis following instructions in the Mini-Prep Cell Manual (Bio-Rad, model 491 Prep Cell, USA, California). Briefly, a 25-mg sample obtained from the AIM was combined with 250 μL electrophoresis sample buffer 4× (0.5 M Tris-HCl pH 6.8, 20% glycerol, 10% SDS, 10% β-mercaptoethanol and 0.05% bromophenol blue) and loaded into a 12% polyacrylamide rod gel, 7 cm high. Electrophoresis was performed under 200-V at room temperature. When the tracking dye front reached the bottom of the gel rod, 100 fractions of 150 μL were collected. Random fractions were loaded into a 16% polyacrylamide SDS-PAGE to locate the protein of interest in the fractions and later delimitate all of those that contained the protein. All fractions containing the protein were pooled, concentrated, and at the same time, electrode buffer eliminated by centrifugal filter 10 000 MW cut-off (Amicon Ultra-4, EMD Millipore, Germany, Darmstadt) at 7,500 ×g, at 4 °C for 10 min, using 30 mM Tris-HCl buffer pH 8.8. Pure protein (100 μL) was sampled in 10 μL aliquots and stored at −20 °C for further analysis. Purified Ps19 concentration was calculated by densitometry as described before.

### Protein MS analysis

Ps19 protein was separated in a 16% SDS-PAGE. After electrophoresis, the gel was stained with Coomassie Blue R-250 and the band was manually excised. The sample was processed at Laboratorio Universitario de Proteómica, UNAM, where it was digested by trypsin (Promega Sequencing Grade Modified Trypsin; Madison, WI, USA) and analyzed by Liquid Chromatograph-Mass Spectrometry (LC-MS). The obtained data were compared with the database at PepBank of Massachusetts General Hospital, GenBank and non-redundant proteins from NCBI.

### Polysaccharide staining and calcium-binding ability on gels

SDS-PAGE 16% polyacrylamide gels were used to study qualitatively putative glycosylations and calcium-binding capability of the Ps19 protein. In particular, saccharide moieties were identified with Periodic Acid-Schiff Stain (PAS) (Sigma-Aldrich, S5133, St. Louis, MO, USA) [37], while calcium-binding ability was tested by cationic carbocyanine dye Stains-All staining [38] (6.8 μg of purified protein were loaded).

### Chitin-binding assay

Chitin-binding assay was performed as described by Montagnani [2]. Briefly, 13.6 μg of purified protein Ps19 or BSA (used as a negative control) were mixed with 100 μL of distilled water and incubated with 1 mg of shrimp shell chitin (Sigma-Aldrich, C9752, St. Louis, MO, USA) under constant stirring for 2 h at 25 °C. The sample was centrifuged for 5 min at 13,000 ×g at room temperature; the supernatant was recovered and stored at 4 °C. The pellet was rinsed thrice with distilled water (200 μL) before washing with 0.2 M NaCl (100 μL), and finally centrifuged for five min at 13,000 ×g; the supernatant was recovered and stored at 4 °C. The pellet and recovered supernatants were mixed with electrophoresis sample buffer 4× (0.5 M Tris-HCl pH 6.8, 20% glycerol, 10% SDS, 10% β-mercaptoethanol and 0.05% bromophenol blue), boiled for 10 min, and loaded into a 16% SDS-PAGE gel. Afterward, proteins were revealed with silver nitrate staining [35] and visualized in a Chemi Doc XRS (Bio-Rad, California, USA).

### *In vitro* CaCO_3_ crystallization in the presence of purified protein

To evaluate the crystal formation of Ps19, it was incubated with three different saturated solutions according to Weiss [39] and Hillner [40]: solution A (40 mM CaCl_2_ pH 8.2, 100 mM NaHCO_3_); solution B (40 mM MgCl_2_ pH 8.2, 100 mM NaHCO_3_) and solution C (100 mM CaCO_3_) these salts by themselves should crystalize calcite and solution B should also form aragonite according to previous reports [41]. Ps19 by itself (6.8 μg of Ps19 in 10 μL and 50 μL of distilled water,) and each solution without the protein (10 μL of distilled water and 50 μL of saturated solution) were used as controls. Each mixture, by triplicate, was incubated over a sterile coverslip inside a Petri dish sealed with parafilm at 4 °C for 30 days. All reagents used are Sigma-Aldrich of molecular biology grade. The morphology of the crystals was examined by Scanning Electron Microscopy (SEM) by triplicate at the Electronic Microscopy Laboratory at Centro de Investigaciones Biológicas del Noroeste (CIBNOR), Mexico.

### Raman

Raman spectroscopy was performed to the *in vitro* obtained crystals by using an InVia micro Raman spectrometer (Renishaw, Nuevo Leon, Mexico) with an excitation line of 532 nm provided by a YAG laser of 100 mW with a spot size of 2 μm x 2 μm. The crystals were scanned for 90 seconds from 100 to 1900 cm^−1^ by triplicate for the specific identification. For all measurements, the slits were set at 200 μm and a 100× objective was used. The analysis was carried out at Laboratorio Nacional de Investigaciones en Nanociencias y Nanotecnología (LINAN)-IPICYT.

## Results

### Isolation and characterization of the Ps19 from the shell of *P*. *sterna*

**Fig 1:**
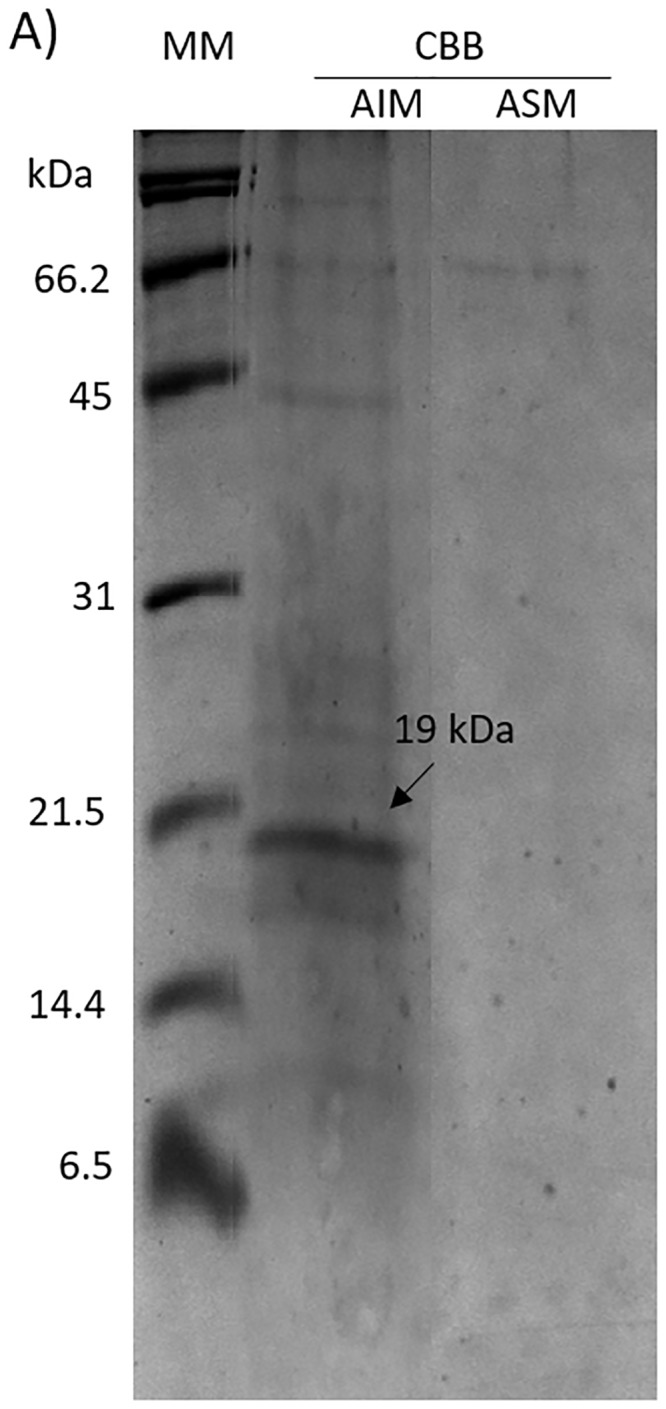
Soluble and insoluble proteins from the shell of *Pteria sterna*. SDS-PAGE 16% polyacrylamide gel. MM: molecular marker, CBB: Coomassie brilliant blue stain; AIM: Acetic-acid insoluble matrix proteins; ASM: Acetic-acid soluble matrix proteins. Arrow indicates the most abundant protein band in the AIM.

The organic matrix from *P*. *sterna* shell was extracted with cold acetic acid. The acid-soluble (ASM) and acid-insoluble (AIM) fractions were separated by electrophoresis and stained with CBB, which showed that AIM had more protein bands than the ASM (Fig 1). The AIM showed an abundant protein band with a relative molecular mass determined from a comparison of relative migration on SDS-PAGE of 19 kDa. The 19kDa protein band, named Ps19, from the AIM was purified in a single step using a preparative electrophoresis. The Ps19 was eluted in the fractions from 58 to 83 (S1 and S2 Figs). This purification procedure yielded a total of 68 μg of Ps19 purified protein from 890 μg of unpurified protein contained in the crude extract of the AIM from *P*. *sterna* (S3 Fig) shell, which had an 18% yield of protein with 85% purity (Table 1).

**Table 1:** Purification of Ps19 from *Pteria sterna* shell.

Species	Step	Total protein (mg)^b^	Target protein (mg)^b^	Yield (%)	Purity (%)
*Pteria sterna*	Crude extract ^a^	0.890	0.369	100	41
	Preparative electrophoresis	0.369	0.075	20	85
	Amicon-washed concentrate	0.068	0.068	18	85

^*a*^ From cold acetic acid extraction of 20 mg of *Pteria sterna* pulverized shell.

^*b*^ Protein concentration determined by image densitometry using Ovalbumin as a standard protein.

**Fig 2:**
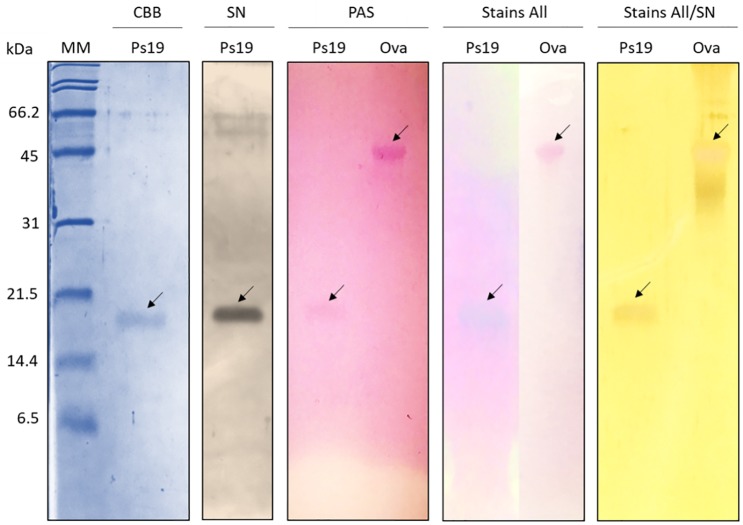
Biochemical characterization of *Pteria sterna* Ps19 protein. *P*. *sterna*, SDS-PAGE 16% polyacrylamide gels. MM: molecular marker, CBB: Coomassie brilliant blue stain, SN: silver nitrate stain, PAS: periodic-acid Schiff stain, Stains All stain, Stains All/SN: silver nitrate stain after Stains All. Ova: ovalbumin used as a positive control for PAS and negative control for Stains-All; Ps19: *Pteria sterna* protein. Arrows indicate positive controls or the protein of interest from each stain.

**Fig 3:**
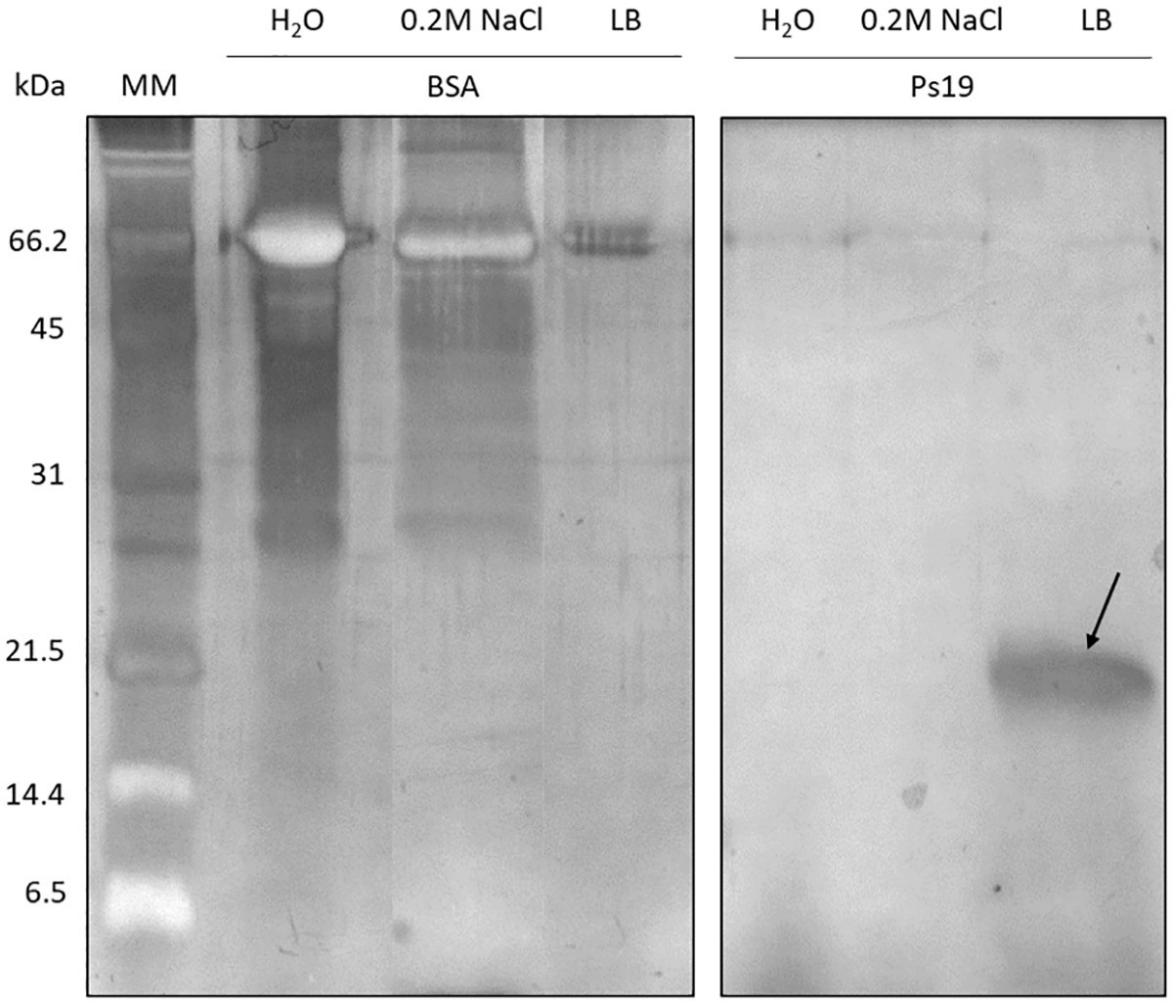
Chitin binding assay from *Pteria sterna* Ps19. SDS-PAGE 16% polyacrylamide stained with silver nitrate. MM: molecular marker; BSA: Bovine serum albumin; Ps19: *P*. *sterna* protein. The arrow indicates Ps19 binding to chitin.

Ps19 is a glycoprotein with acid amino acid residues with potential calcium-binding properties according to PAS and Stain All/silver staining (Fig 2). The chitin-binding capability of Ps19 was analyzed by incubation of the isolated protein with chitin and successive washing solutions. Ps19 was clearly present in the pellet treated with Laemmli buffer indicating that Ps19 is capable of binding α-chitin unlike BSA (negative control) that is not present in the pellet (containing α-chitin), but in the aqueous fraction of the washes (Fig 3).

### Protein sequencing

The Ps19 was sequenced *de novo* by Liquid Chromatography-Mass Spectrometry, its amino acid composition indicated the presence of a high proportion of Leu (17%), Asp (11%), Ser (8%) and Ala (8%); thus, it is potentially an acidic protein with a 17% of acidic residues (Asp/Glu). Using the Basic Local Alignment Search Tool for proteins (BLASTp) against non-redundant and Mollusca proteins database with a cutoff of 0.3 for the E-value and using as query sequence the peptides obtained by Mass Spectrometry, the Ps19 match with two uncharacterized proteins from *Crassostrea gigas* with 89 and 78% of identity and 2.1 and 1.0 E-values respectively, with two proteins from *Mizuhopecten yessoensis* with also 89 and 78% of identity and 3.0 and 0.5 E-value respectively. Other peptides showed 67% identity with a hypothetical protein from *Lottia gigantea* and 100% identity with a BTB/POZ domain, ankyrin repeat from *Aplysia californica* with 100% identity and 0.5 E-value. Finally, the last peptide had 80% similarity with 0.4 E-value to a serine beta-lactamase-like protein from *Biomphalaria glabrata* and 64% similarity with a hypothetical protein from *Elysia clorotica* and 2.5 E-value (Table 2).

**Table 2:** BLAST hits of Ps19 peptides against non-redundant and Mollusca proteins.

No.	Sequence	Precursor (Da)	MW	BLAST hits	E value	Identity (%)	Accession (ID)
1	DLCDAGAVR	488.7	975.4	-	-	-	-
2	MDALGLANR	481.2	960.5	-	-	-	-
3	YACVQCR	478.7	955.4	-	-	-	-
4	DVTAVTWVADDLFP	798.9	1595.8	Uncharacterized protein (*Crassostrea virginica*)	2.1	89	XP_022293857.1
				Uncharacterized protein (*Mizuhopecten yessoensis*)	3.0	89	XP_021371593.1
5	PFVDDQYCYVLK	852.9	1703.8	Uncharacterized protein (*Crassostrea virginica*)	1.0	78	XP_009057105.1
				Hypothetical protein (*Lottia gigantea*)	1.0	67	XP_009057105.1
6	DLSTDSTFLWALK	840.9	1679.9	-	-	-	-
7	LWTQYNSNLCSAQLR	903.7	1805.4	-	-	-	-
8	GELNDEFSTCNSPR	979.9	1957.8	-	-	-	-
9	DGDVLMWALR	656.8	1311.6	BTB/POZ domain, ankyrin repeat (*Aplysia californica*)	0.5	100	XP_005100573.1
				Uncharacterized protein (*Mizuhopecten yessoensis*)	0.5	78	XP_021377093.1
10	YSLDPLSCS	924.1	1846.8	-	-	-	-
11	FPGSPYVELYKWR	967.4	1932.9	Serine beta-lactamase-like protein (*Biomphalaria glabrata*)	0.4	80	XP_013086113.1
				Hypothetical protein (*Elysia chlorotica*)	2.5	64	RUS87181.1

BLAST analysis using as query sequence the peptides obtained by Mass Spectrometry against non-redundant and Mollusca proteins database with a cutoff of 3 for the E-value.

### *In vitro* CaCO_3_ crystallization assay

**Fig 4:**
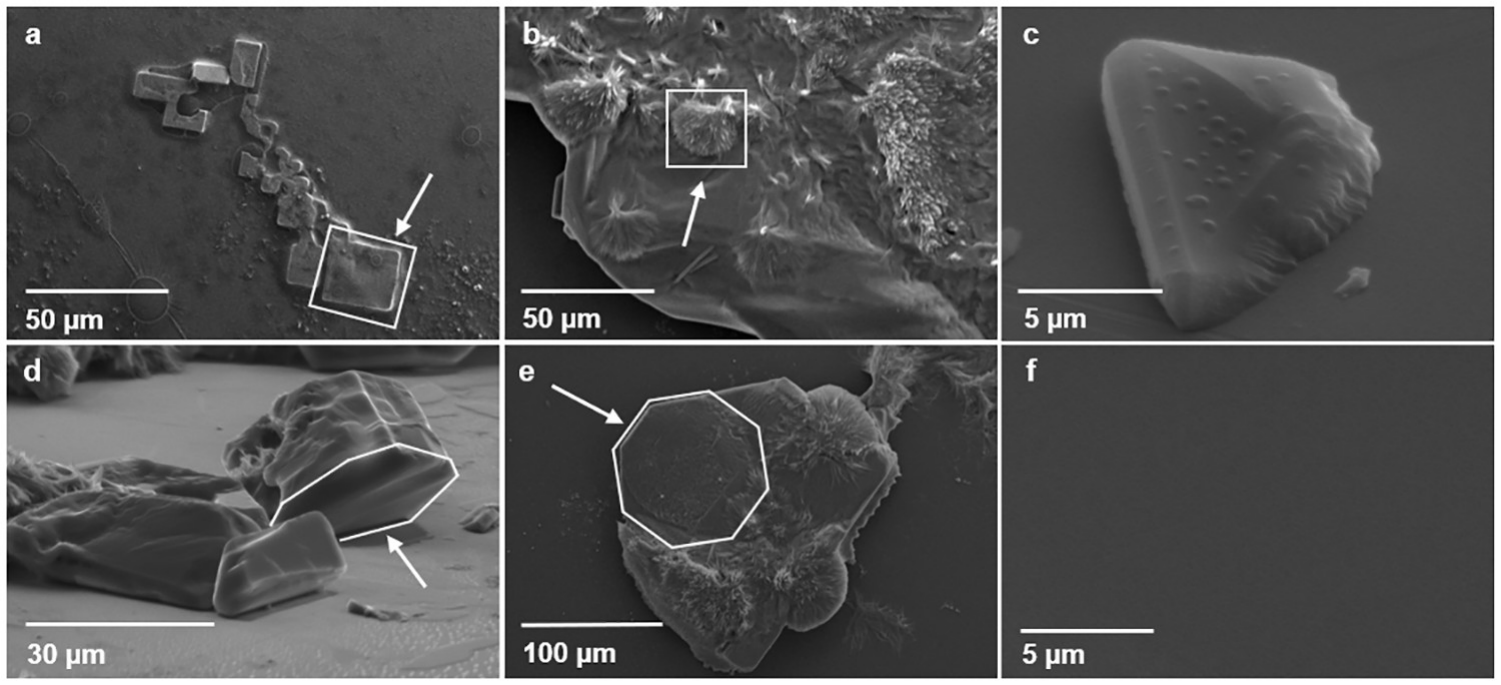
Effect of purified Ps19 on CaCO_3_ crystal growth with different salt solutions. a: 40 mM CaCl_2_ pH 8.2, 100 mM NaHCO_3_ (solution A); b: 40 mM MgCl_2_ pH 8.2, 100 mM NaHCO_3_ (solution B); c: 100 mM CaCO_3_ (solution C). d, e, and f: Ps19 with solutions A, B, and C, respectively. Arrows indicate calcite crystals in a, aragonite crystals in b, and aragonite plates in d and e. The geometrical structures for each case have been delimited in white.

Crystalline CaCO_3_ formation was evaluated by SEM when no protein was added to solution A (40 mM CaCl_2_ pH 8.2, 100 mM NaHCO_3_) and solution C (100 mM CaCO_3_) calcite crystals were observed, sizing 25 μm for solution A and 20 μm for solution C. These crystals showed the typical geometry of calcite, with smooth faces and sharp angles (Fig 4a and 4c). Solution B (40 mM MgCl_2_ pH 8.2, 100 mM NaHCO_3_), displayed aragonite crystals, with a needle-like morphology and variable sizing (Fig 4b). When Ps19 was added to solution A and solution B, aragonite plates were appreciated (Fig 4d and 4e), the solution containing CaCl_2_ (solution A) formed 30 μm sizing plates (Fig 4d), while solution B (containing MgCl_2_) formed plates of 100 μm size (Fig 4e). Both crystal structures formed well defined octagonal shaped plates. However, no crystals were formed when solution C (100 mM CaCO_3_) was incubated with Ps19 (Fig 4f).

### Raman analysis

**Fig 5:**
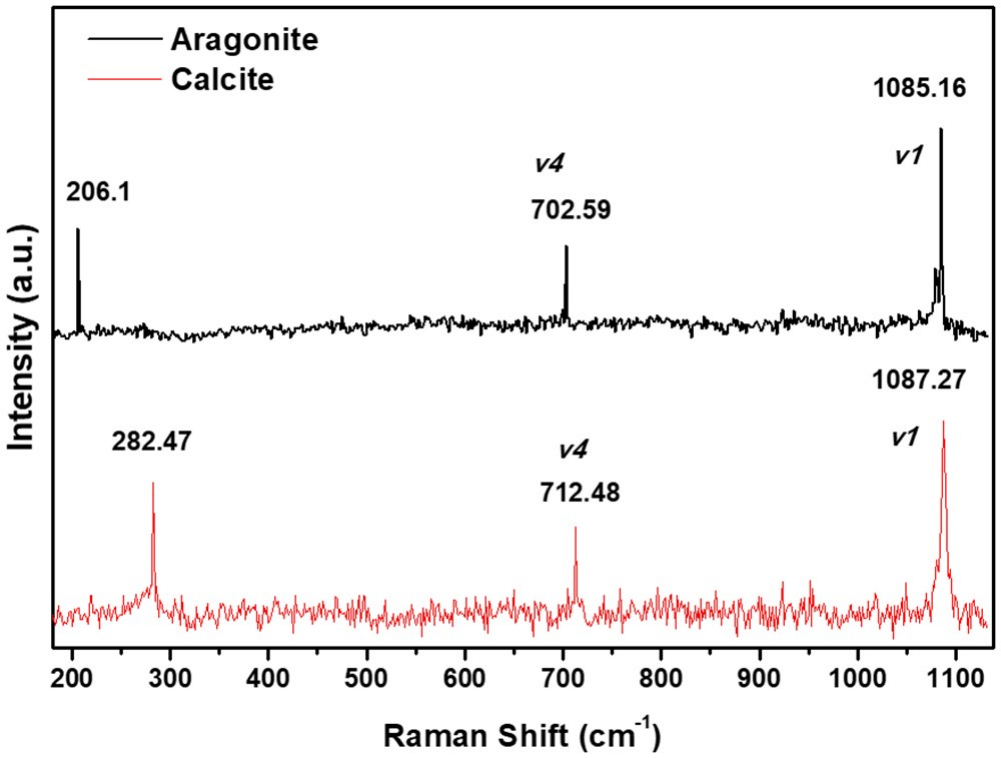
Raman spectra of calcite crystals from controls and aragonite crystals induced by the Ps19 protein isolated from the acetic acid-insoluble matrix from *Pteria sterna* shell.

Raman spectroscopy was used to confirm the identity of calcite and aragonite crystal formation. The spectra for the samples corresponding to Fig 4a and 4e are shown in Fig 5. Usually, calcite crystals contain two CaCO_3_ units, for a total of ten atoms; its nucleation and crystal growth have been extensively studied by Raman spectroscopy and theoretical calculations [42]. The presence of the active vibrational modes for Raman frequencies at 282.47 cm^-1^ (translatory oscillations of CO_3_ groups), 712.48 cm^-1^ (*v*4, asymmetric bending), and 1087.27 cm^-1^ (*v*1, symmetric stretching of CO_3_ groups), confirms the correct formation of the rhombohedral primitive cell of calcite with space group $D_{3d}^{6}(R\overset{-}{3}C)$ and parameters of a = 5.03 and c = 17.325 Å, respectively [43]. On the other hand, the Raman spectrum for the aragonite shows similar vibrations modes than calcite, but with a slight shift to lower energies for all modes, according to its orthorhombic primitive cell. The crystal contains four CaCO_3_ units, for a total of twenty atoms; the presence of the Raman-active modes at 206.1 cm^-1^, 702.595 cm^-1^, and 1085.16 cm^-1^, has been reported for aragonite crystalline powder and confirms the correct formation of the orthorhombic lattice with space group $D_{2h}^{16}(Pnma)$ and cell parameters of 5.008, 8.029, and 5.861 Å for the a, b, and c axes, respectively [44].

## Discussion

A novel shell matrix protein from the acetic acid-insoluble matrix from *P*. *sterna* shell was isolated and characterized. Previous studies have revealed that acetic acid insoluble shell matrix proteins have Asp and Glu residues in high proportion turning them into acidic proteins, which make their isolation a challenging process. Ps19 showed a high proportion of these residues (17%); therefore it may have been an acidic protein and its isolation was difficult, having low yield (18%) extraction but high purity (85%). Acetic acid-insoluble proteins from other species have been extracted and an attempt to describe and characterize them all has been done, and although some have been described, many still remain to be characterized; their role in shell biomineralization is still unknown [12,28,45].

Biochemical assays for Ps19 have shown it has chitin-binding abilities, which is a vital characteristic for interactions between shell matrix proteins and organic biomolecules to create a frame for calcium carbonate mineralization [5,26,46,47]. β-chitin is one of the main organic components of interlamellar nacre plates, and it is also considered as a possible nucleation site for aragonite sheets due to its interaction with other matrix proteins [48–50]. According to this evidence, Ps19 may not be only involved in aragonite plate mineralization but also in its nucleation. Proteins like Pif-like and hichin (AIM proteins) are known for having chitin-binding conserved domains rich in Gly and Cys residues [27,30], but according to BLAST analysis, neither of these known domains were found in the peptide sequence of Ps19; however, it poses this capability *in vitro*, suggesting other unknown chitin-binding domains might exist. The Pearlin/N16 family does not have a defined chitin-binding domain; nonetheless, the biochemical analysis showed they pose chitin-binding ability [2,32] coinciding with Ps19 results.

Previous reports have shown that proteins, such as N14, N16, MSI60 have specific GXN domains [31–33] or D-rich domains as Aspein [24], which are involved in calcium-binding in *Pinctada fucata* [51]. Ps19 showed calcium-binding capability, but its amino acid sequence did not suggest the presence of any conserved domain according to the BLAST analysis with a 0.1 cutoff E-value; however, when this value was broadened to 3.0, it showed two hits with 0.5 E-value for DGDVLMWALR, one with 100% identity for a BTB/POZ domain of *Aplysia californica* which is a domain found in different proteins related to cytoskeletal arrengment, regulation of animal transcription, and other functions involving protein-protein interactions [52], and the second to a *Mizuhopecten yessoensis* uncharacterized protein with 78% similarity. The similarity with the BTB/POZ domain may indicate Ps19 is a protein involved in protein-protein interactions, perhaps to help in the aragonite plate formation, what kind of protein-protein interactions it is involved it is yet to be studied. Another hit, with 0.4 E-value for FPGSPYVELYKWR peptide, showed 80% similarity with a serine beta-lactamase-like protein from *Biomphalaria glabrata*. Beta-lactamase is a mitochondrial protein related to antibiotic resistance with enzymatic activity [53,54], so further analysis of Ps19 is needed to prove if it has enzymatic activity or at least share some characteristics with this family of proteins. The other hits showed similarity with uncharacterized proteins or with hypothetical proteins, so their function is not well described and more information is required to make an statement.

Glycosylation is an important feature for SMPs because it imparts an acidic pH to the protein [55]. It has been proposed that acidic sugars, especially sulfated, may concentrate calcium ions at the vicinity of the acidic proteins, inducing crystal nucleation [56]. They may also play an important role in mineral surface recognition and in polymorph selection [55–57]. Ps19 showed glycosylation, which may be an acidic protein; therefore, its glycosylation may be responsible for its capacity to crystalize aragonite plates *in vitro* despite the salt it is incubated with.

Saturated solutions have demonstrated to contain -HCO_3_, Na^2+^ or Ca^2+^ that crystalize calcite crystals while incubating with Mg^+2^ will form aragonite crystals; when shell matrix proteins are aggregated to this solutions, they might induce calcite or aragonite crystals respectively [40,41]. Solutions forming calcite or aragonite crystals were consistent with the reported results in this research; however, Ps19 is capable of inducing aragonite crystals despite the salt it is incubated with (solution A or B), indicating it selectively induces aragonite plates similar to nacre plates *in vitro*. No crystal formation was observed when Ps19 was incubated with solution C (100 mM CaCO_3_), which might be associated to protein concentration as previously reported by other authors. This result suggests that high protein concentration may inhibit calcium carbonate crystal formation, acting as a nucleating regulator [39,58,59].

Calcium carbonate polymorph selection does not only depend on the salt composition from the incubation solution; under experimental conditions, aragonite crystals are easier to form on confined spaces (> 25 nm diameter tubes), but in nature such conditions do not exist. Mollusk nacre plates are greater than 25 nm diameter due to matrix shell protein interactions between minerals and fibroin-like framework molecules [60]. In this study, Ps19 was capable of binding chitin and Ca^2+^ ions although according to the partial peptide sequence, it did not show any known domain associated to these characteristics, such as GN- repeat or GYS motifs, which have been observed in most matrix shell proteins [1,2,27,31,32]. As other SMPs, Ps19 binds polysaccharides and calcium ions, which are necessary for mineralizing calcium carbonate; therefore, Ps19 acts as an intermediary between calcium carbonate crystals and the organic matrix; moreover, it might function as an aragonite nucleation template according to *in vitro* crystallization assays although further studies are needed to corroborate this role in mineralization. These characteristics have demonstrate the importance of Ps19 in shell biomineralization.

These evidences highlight that despite Ps19 did not show any chitin-binding conserved domain, it can bind chitin and interact with Ca^2+^ to nucleate aragonite plates *in vitro*. Therefore, other chitin-binding sequences must exist, as well as other calcium-binding domains. Similarity with a BTB/POZ domain may suggest Ps19 has an specific domain for interacting with other proteins involved in biomineralization, also, similarity with a serine beta-lactamase-like protein suggest it may have enzymatic activity, but this is yet to be tested.

## Conclusions

This study described the biochemical characteristics of Ps19 from the shell of bivalve *Pteria sterna*. Ps19 is a glycoprotein with calcium and chitin-binding capability, which are important characteristics for aragonite nucleation. Ps19 is capable of cristallizing calcium carbonate *in vitro* by forming aragonite plates that are necessary to structure nacre. Further research must be performed to obtain the full protein sequence of Ps19 to characterize its structure and understand its potential interaction with calcium and chitin molecules to form nacre in mollusk shells.

## Supporting information

S1 Fig Relative mobility of Ps19 in the acetic-acid insoluble matrix (AIM) proteins from *P*. *sterna* SDS-PAGE 16% polyacrylamide. The axes represent the relative mobility (Rf) of proteins and the logarithm of their molecular weight (log MW). MWS: molecular weight standards (Bio-Rad 1610317).
(TIF)

S2 Fig SDS-PAGE 16% polyacrylamide from the preparative electrophoresis from *Pteria sterna* stained with silver nitrate. MM: molecular marker; 1–9: random samples of 76–150 fractions. The arrow indicates the fraction containing the protein of interest.
(TIF)

S3 Fig Ovalbumin standard curve. (A) SDS-PAGE 16% polyacrylamide gel. Ovalbumin standard curve indicated by an arrow (8.0–0.25 μg·μL^-1^) stained with CBB for pixel density determination to calculate the linear equation and quantify protein bands. MM: molecular marker; Ovalbumin concentrations (μg·μL^-1^). (B) Ovalbumin standard curve graphic. The axes represent pixel volume and protein quantity (μg). Circles represent Ovalbumin (μg), the square corresponds to purified *Pteria sterna* protein.
(TIF)

S1 Raw image (PDF)

## References

[1] Matsushiro A, Miyashita T, Miyamoto H, Morimoto K, Tonomura B, Tanaka A, et al Presence of Protein Complex is Prerequisite for Aragonite Crystallization in the Nacreous Layer. Mar Biotechnol (NY). 2003;5: 37–44. 10.1007/s10126-002-0048-3 12925917

[2] Montagnani C, Marie B, Marin F, Belliard C, Riquet F, Tayalé A, et al Pmarg-Pearlin is a Matrix Protein Involved in Nacre Framework Formation in the Pearl Oyster Pinctada margaritifera. ChemBioChem. 2011;12: 2033–2043. 10.1002/cbic.201100216 21796751

[3] Miyashita T, Takagi R, Okushima M, Nakano S, Miyamoto H, Nishikawa E, et al Complementary DNA Cloning and Characterization of Pearlin, a New Class of Matrix Protein in the Nacreous Layer of Oyster Pearls. Mar Biotechnol (NY). 2000;2: 409–418. Available: http://www.ncbi.nlm.nih.gov/pubmed/11246407 11246407 10.1007/pl00021687

[4] Barthelat F. Nacre from mollusk shells: a model for high-performance structural materials. IOP Publ. 2010;5: 1–8. 10.1088/1748-3182/5/3/035001 20729573

[5] Marin F, Le Roy N, Marie B. The formation and mineralization of mollusk shell. Front Biosci. 2012; 1099–1125. 10.2741/s321 22202112

[6] Tushtev K, Murck M, Grathwohl G. On the nature of the stiffness of nacre. Mater Sci Eng C. 2008;28: 1164–1172. 10.1016/j.msec.2007.10.039

[7] Rousseau M, Meibom A, Gèze M, Bourrat X, Angellier M, Lopez E. Dynamics of sheet nacre formation in bivalves. J Struct Biol. Academic Press; 2009;165: 190–195. 10.1016/J.JSB.2008.11.011 19121399

[8] Addadi L, Joester D, Nudelman F, Weiner S. Mollusk Shell Formation: A Source of New Concepts for Understanding Biomineralization Processes. Chem Eur J. 2006;12: 980–987. 10.1002/chem.200500980 16315200

[9] Levi-Kalisman Y, Falini G, Addadi L, Weiner S. Structure of the Nacreous Organic Matrix of a Bivalve Mollusk Shell Examined in the Hydrated State Using Cryo-TEM. J Struct Biol. 2001;135: 8–17. 10.1006/jsbi.2001.4372 11562161

[10] Belcher AM, Wu XH, Christensen RJ, Hansma P. K., Stucky GD, Morse DE. Control of crystal phase switching and orientation by soluble mollusc-shell proteins. Nature. 1996;381: 56–58.

[11] Marie B, Arivalagan J, Berland S, Marie A, Bolbach G, Berland S, et al Deep conservation of bivalve nacre proteins highlighted by shell matrix proteomics of the Unionoida Elliptio complanata and Villosa lienosa. J R Soc Interface. 2016;14: 1–11. 10.1098/rsif.2016.0846 PMC5310735 28123096

[12] Marie B, Arivalagan J, Dubost L, Berland S, Marie A, Marin F. Unveiling the evolution of bivalve nacre proteins by shell proteomics of unionoidae. Key Eng Mater. 2015;672: 158–167. doi: www.scientific.net/KEM.672.158

[13] Evans JS. “Tuning in” to Mollusk Shell Nacre- and prismatic-associated protein terminal sequences. Implications for biomineralization and the construction of high performance inorganic—Organic composites. Chem Rev. 2008;108: 4455–4462. 10.1021/cr078251e 18793025

[14] Marin F, Luquet G, Marie B, Medakovic D. Molluscan Shell Proteins: Primary Structure, Origin, and Evolution. Curr Top Dev Biol. 2007;80: 209–276. 10.1016/S0070-2153(07)80006-8 17950376

[15] Marin F, Marie B, Ben Hamada S, Silva P, Le Roy N, Wolf SE, et al “Shellome”: proteins involved in mollusc shell biomineralization–diversity, functions. Recent Adv Pearl Res. 2013; 149–166. Available: http://hal.archives-ouvertes.fr/hal-00793668/

[16] Du J, Liu C, Xu G, Xie J, Xie L, Zhang R. fam20C participates in the shell formation in the pearl oyster, Pinctada fucata. Sci Rep. 2018;8 10.1038/s41598-018-21797-w 29476076 PMC5824888

[17] Zhang C, Zhang R. Matrix proteins in the outer shells of molluscs. Mar Biotechnol. 2006;8: 572–586. 10.1007/s10126-005-6029-6 16614870

[18] Mann K, Edsinger-gonzales E, Mann M. In-depth proteomic analysis of a mollusc shell: acid-soluble and acid-insoluble matrix of the limpet Lottia gigantea. Proteome Sci. 2012;10: 1–18. 22540284 10.1186/1477-5956-10-28 PMC3374290

[19] Zhang C, Xie L, Huang J, Liu X, Zhang R. A novel matrix protein family participating in the prismatic layer framework formation of pearl oyster, Pinctada fucata. Biochem Biophys Res Commun. Academic Press; 2006;344: 735–740. 10.1016/J.BBRC.2006.03.179 16630535

[20] Caiping M, Cen Z, Yancheng N, Liping X, Zhang R. Extraction and Purification of Matrix Protein from the Nacre of Pearl Oyster Pinctada fucata. Tsinghua Sci Technol. 2005;10: 499–503.

[21] Yang D, Yan Y, Yang X, Liu J, Zheng G, Xie L, et al A basic protein, N25, from a mollusk modifies calcium carbonate morphology and shell biomineralization. J Biol Chem. 2019;294: 8371–8383. 10.1074/jbc.RA118.007338 30967473 PMC6544838

[22] Wang SN, Yan XH, Wang R, Yu DH, Wang XX. A microstructural study of individual nacre tablet of Pinctada maxima. J Struct Biol. Elsevier Inc.; 2013;183: 404–411. 10.1016/j.jsb.2013.07.013 23933393

[23] Mouries LP, Almeida M-J, Milet C, Berland S, Lopez E. Bioactivity of nacre water-soluble organic matrix from the bivalve mollusk Pinctada maxima in three mammalian cell types: fibroblasts, bone marrow stromal cells and osteoblasts. Comp Biochem Physiol Part B Biochem Mol Biol. 2002;132: 217–229. 10.1016/s1096-4959(01)00524-3 11997223

[24] Tsukamoto D, Sarashina I, Endo K. Structure and expression of an unusually acidic matrix protein of pearl oyster shells. Biochem Biophys Res Commun. 2004;320: 1175–1180. 10.1016/j.bbrc.2004.06.072 15249213

[25] Ma Y, Berland S, Andrieu J, Feng Q, Bédouet L. What is the difference in organic matrix of aragonite vs. vaterite polymorph in natural shell and pearl? Study of the pearl-forming freshwater bivalve mollusc Hyriopsis cumingii. Mater Sci Eng C. 2013;33: 1521–1529. 10.1016/j.msec.2012.12.057 23827604

[26] Marie B, Luquet G, Bédouet L, Milet C, Guichard N, Medakovic D, et al Nacre calcification in the freshwater mussel Unio pictorum: Carbonic anhydrase activity and purification of a 95 kDa calcium-binding glycoprotein. ChemBioChem. 2008;9: 2515–2523. 10.1002/cbic.200800159 18810748

[27] Feng D, Li Q, Yu H, Kong L, Du S. Identification of conserved proteins from diverse shell matrix proteome in Crassostrea gigas: Characterization of genetic bases regulating shell formation. Sci Rep. 2017;7: 1–12. 28374770 10.1038/srep45754 PMC5379566

[28] Marie B, Zanella-Cléon I, Le Roy N, Becchi M, Luquet G, Marin F. Proteomic analysis of the acid-soluble nacre matrix of the bivalve unio pictorum: Detection of novel carbonic anhydrase and putative protease inhibitor proteins. ChemBioChem. 2010;11: 2138–2147. 10.1002/cbic.201000276 20815006

[29] Jin C, Zhao J, Pu J, Liu X, Li J. Hichin, a chitin binding protein is essential for the self-assembly of organic frameworks and calcium carbonate during shell formation. Int J Biol Macromol. Elsevier B.V.; 2019;135: 745–751. 10.1016/j.ijbiomac.2019.05.205 31152837

[30] Gao P, Liao Z, Wang XX, Bao LF, Fan MH, Li XM, et al Layer-by-layer proteomic analysis of mytilus galloprovincialis shell. PLoS One. 2015;10: 1–19. 10.1371/journal.pone.0133913 26218932 PMC4517812

[31] Kono M, Hayashi N, Samata T. Molecular mechanism of the nacreous layer formation in Pinctada maxima. Biochem Biophys Res Commun. 2000;269: 213–218. 10.1006/bbrc.2000.2274 10694502

[32] Samata T, Hayashi N, Kono M, Hasegawa K, Horita C, Akera S. A new matrix protein family related to the nacreous layer formation of Pinctada fucata. FEBS Lett. 1999;462: 225–229. 10.1016/s0014-5793(99)01387-3 10580124

[33] Sudo S, Fujikawa T, Nagakura T, Ohkubo T, Sakaguchi K, Tanaka M, et al Structures of mollusc shell framework proteins. Nature. 1997;387: 563–564. 10.1038/42391 9177341

[34] Laemmli UK. Cleavage of structural proteins during the assembly of the head of bacteriophage T4. Nature. 1970;227: 680–685. 10.1038/227680a0 5432063

[35] Rabilloud T, Vuillard L, Gilly C, Lawrence JJ. Silver-staining of proteins in polyacrylamide gels: a general overview. Cell Mol Biol. 1994;40: 57–75. 8003936

[36] Righetti PG, Chillemi F. Isoelectric focusing of peptides. J Chromatogr A. Elsevier; 1978;157: 243–251. 10.1016/S0021-9673(00)92339-2

[37] Zacharius RM, Zell TE, Morrison JH, Woodlock JJ. Glycoprotein staining following electrophoresis on acrylamide gels. Anal Biochem. Academic Press; 1969;30: 148–152. 10.1016/0003-2697(69)90383-2 4183001

[38] Green MR, Pastewka J V., Peacock AC. Differential staining of phosphoproteins on polyacrylamide gels with a cationic carbocyanine dye. Anal Biochem. 1973;56: 43–51. 10.1016/0003-2697(73)90167-x 4128675

[39] Weiss IM, Kaufmann S, Mann K, Fritz M. Purification and characterization of perlucin and perlustrin, two new proteins from the shell of the mollusc Haliotis laevigata. Biochem Biophys Res Commun. 2000;267: 17–21. 10.1006/bbrc.1999.1907 10623567

[40] Hillner PE, Gratz AJ, Manne S, Hansma PK. Atomic-Scale Imaging of Calcite Growth and Dissolution in Real-Time. Geology. 1992;20: 359–362.

[41] Fricke M, Volkmer D. CrystallizationofCalciumCarbonateBeneath Insoluble Monolayers: Suitable Models of Mineral–Matrix Interactions in Biomineralization? Top Curr Chem. 2007;270: 1–41.

[42] De La Pierre M, Carteret C, Maschio L, André E, Orlando R, Dovesi R. The Raman spectrum of CaCO3polymorphs calcite and aragonite: A combined experimental and computational study. J Chem Phys. 2014;140: 10.1063/1.4871900 24784289

[43] Rutt HN, Nicola JH. Raman spectra of carbonates of calcite structure. J Phys C Solid State Phys. 1974;7: 4522–4528. 10.1088/0022-3719/7/24/015

[44] Urmos J, Sharma F, Mackenzie FT. Characterization of some biogenic carbonates with Raman spectroscopy. Am Mineral. 1991;76: 641–646.

[45] Liao Z, Jiang Yting, Sun Q, Fan Mhua, Wang Jxin, Liang Hying. Microstructure and in-depth proteomic analysis of Perna viridis shell. PLoS ONE. 2019 10.1371/journal.pone.0219699 31323046 PMC6641155

[46] Baqueiro E, Aldana D. Mecanismo de formacion de conchas de moluscos. Av y Perspect. 1995;14: 231–236.

[47] Nudelman F. Seminars in Cell & Developmental Biology Nacre biomineralisation: A review on the mechanisms of crystal nucleation. Semin Cell Dev Biol. Elsevier Ltd; 2015;46: 2–10. 10.1016/j.semcdb.2015.07.004 26205040

[48] Addadi L, Joester D, Nudelman F, Weiner S. Mollusk Shell Formation: A Source of New Concepts for Understanding Biomineralization Processes. Chem—A Eur J. 2006;12: 980–987. 10.1002/chem.200500980 16315200

[49] Keene EC, Evans JS, Estroff LA. Silk Fibroin Hydrogels Coupled with the n16N−β-Chitin Complex: An in Vitro Organic Matrix for Controlling Calcium Carbonate Mineralization. Cryst Growth Des. 2010;10: 5169–5175. 10.1021/cg1009303

[50] Bahn SY, Jo BH, Hwang BH, Choi YS, Cha HJ. Role of Pif97 in Nacre Biomineralization: In Vitro Characterization of Recombinant Pif97 as a Framework Protein for the Association of Organic − Inorganic Layers in Nacre. Cryst Growth Des. 2015;15: 3666–3673. 10.1021/acs.cgd.5b00275

[51] Sato Y, Inoue N, Ishikawa T, Ishibashi R, Obata M, Aoki H, et al Pearl Microstructure and Expression of Shell Matrix Protein Genes MSI31 and MSI60 in the Pearl Sac Epithelium of Pinctada fucata by In Situ Hybridization. PLoS One. 2013;8 10.1371/journal.pone.0052372 23341897 PMC3544836

[52] Aravind L, Koonin E V. Fold prediction and evolutionary analysis of the POZ domain: Structural and evolutionary relationship with the potassium channel tetramerization domain. J Mol Biol. 1999;285: 1353–1361. 10.1006/jmbi.1998.2394 9917379

[53] Fróes AM, da Mota FF, Cuadrat RRC, Dávila AMR. Distribution and classification of serine β-lactamases in Brazilian hospital sewage and other environmental metagenomes deposited in public databases. Front Microbiol. 2016;7: 1–15. 27895627 10.3389/fmicb.2016.01790 PMC5108929

[54] Keckesova Z, Donaher JL, De Cock J, Freinkman E, Lingrell S, Bachovchin DA, et al LACTB is a tummor suppressor that modulates lipid metabolism and cell state. Nature. 2017;543: 681–686. 10.1038/nature21408 28329758 PMC6246920

[55] Samata T, Samata T. CA-BINDING GLYCOPROTEINS IN MOLLUSCAN SHELLS WITH DIFFERENT TYPES OF ULTRASTRUCTURE. The veliger. 1990;33: 190–201. Available: https://www.biodiversitylibrary.org/part/94225

[56] Marie B, Luquet G, De Barros JP, Guichard N, Morel S. The shell matrix of the freshwater mussel Unio pictorum (Paleoheterodonta, Unionoida) Involvement of acidic polysaccharides from glycoproteins in nacre mineralization. FEBS J. 2007;274: 2933–2945. 10.1111/j.1742-4658.2007.05825.x 17488282

[57] Levi Y, Albeck S, Brack A, Weiner S, Addadi L. Control over aragonite crystal nucleation and growth: An in vitro study of biomineralization. Chem—A Eur J. 1998;4: 389–396. 10.1002/(SICI)1521-3765(19980310)4:3<389::AID-CHEM389>3.0.CO;2-X

[58] Chen Y, Gao J, Xie J, Liang J, Zheng G, Xie L, et al Transcriptional regulation of the matrix protein Shematrin-2 during shell formation in pearl oyster. J Biol Chem. 2018;293: 17803–17816. 10.1074/jbc.RA118.005281 30282805 PMC6240880

[59] Zhang R, Xie L, Yan Z. Biomineralization mechanism of the pearl oyster, pinctada fucata. Biomineralization Mechanism of the Pearl Oyster, Pinctada Fucata. 2018 10.1007/978-981-13-1459-9

[60] Zeng M, Kim Y-Y, Anduix-Canto C, Frontera C, Laundy D, Kapur N, et al Confinement generates single-crystal aragonite rods at room temperature. Proc Natl Acad Sci. 2018. 10.1073/pnas.1718926115 PMC6065038 29967143

