# Supplementary figures and images for: Ps19, a novel chitin binding protein from Pteria sterna capable to mineralize aragonite plates in vitro
Source: PLoS One. 2020 Mar 19;15(3):e0230431. doi: 10.1371/journal.pone.0230431 (PMC7081993; doi:10.1371/journal.pone.0230431)

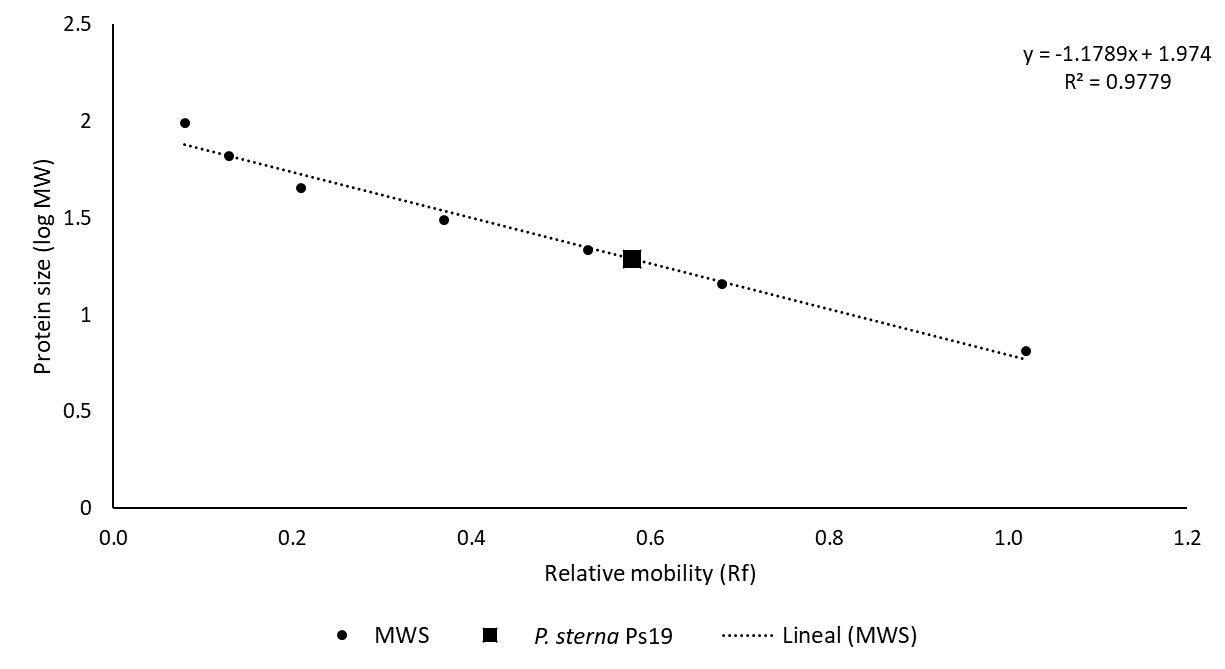

Supplement: S1 Fig — The axes represent the relative mobility (Rf) of proteins and the logarithm of their molecular weight (log MW). MWS: molecular weight standards (Bio-Rad 1610317). (TIF) [file pone.0230431.s001.tif]

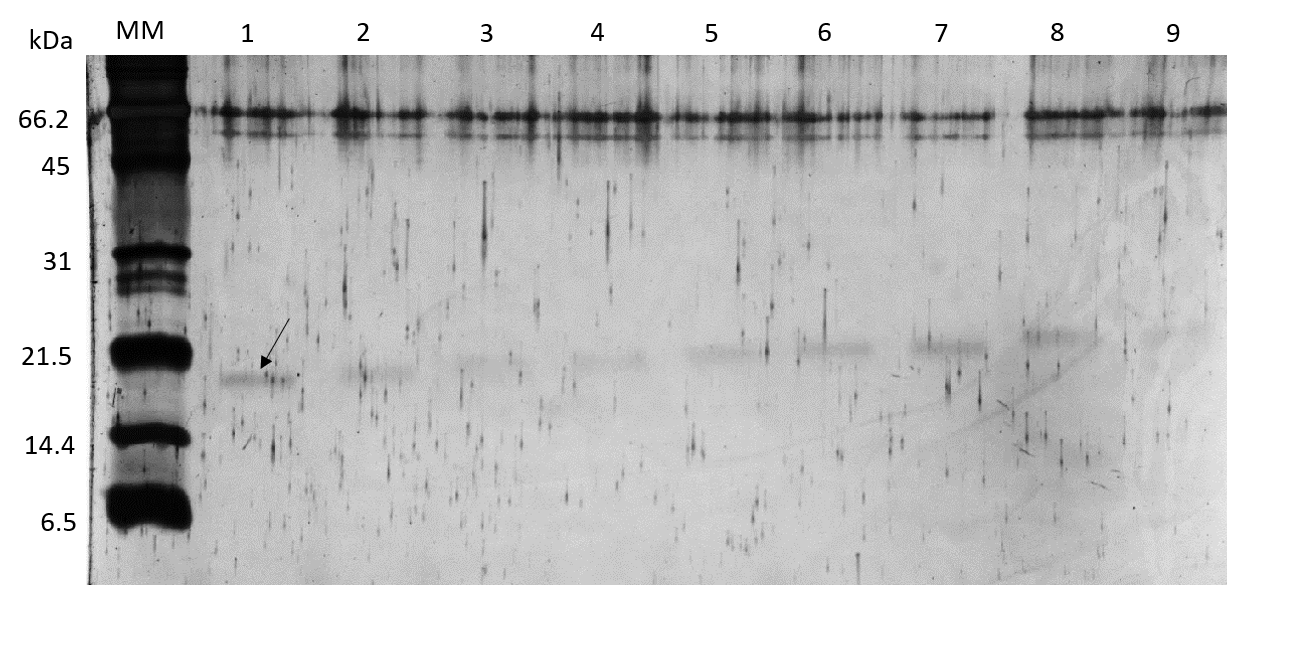

Supplement: S2 Fig — MM: molecular marker; 1–9: random samples of 76–150 fractions. The arrow indicates the fraction containing the protein of interest. (TIF) [file pone.0230431.s002.tif]

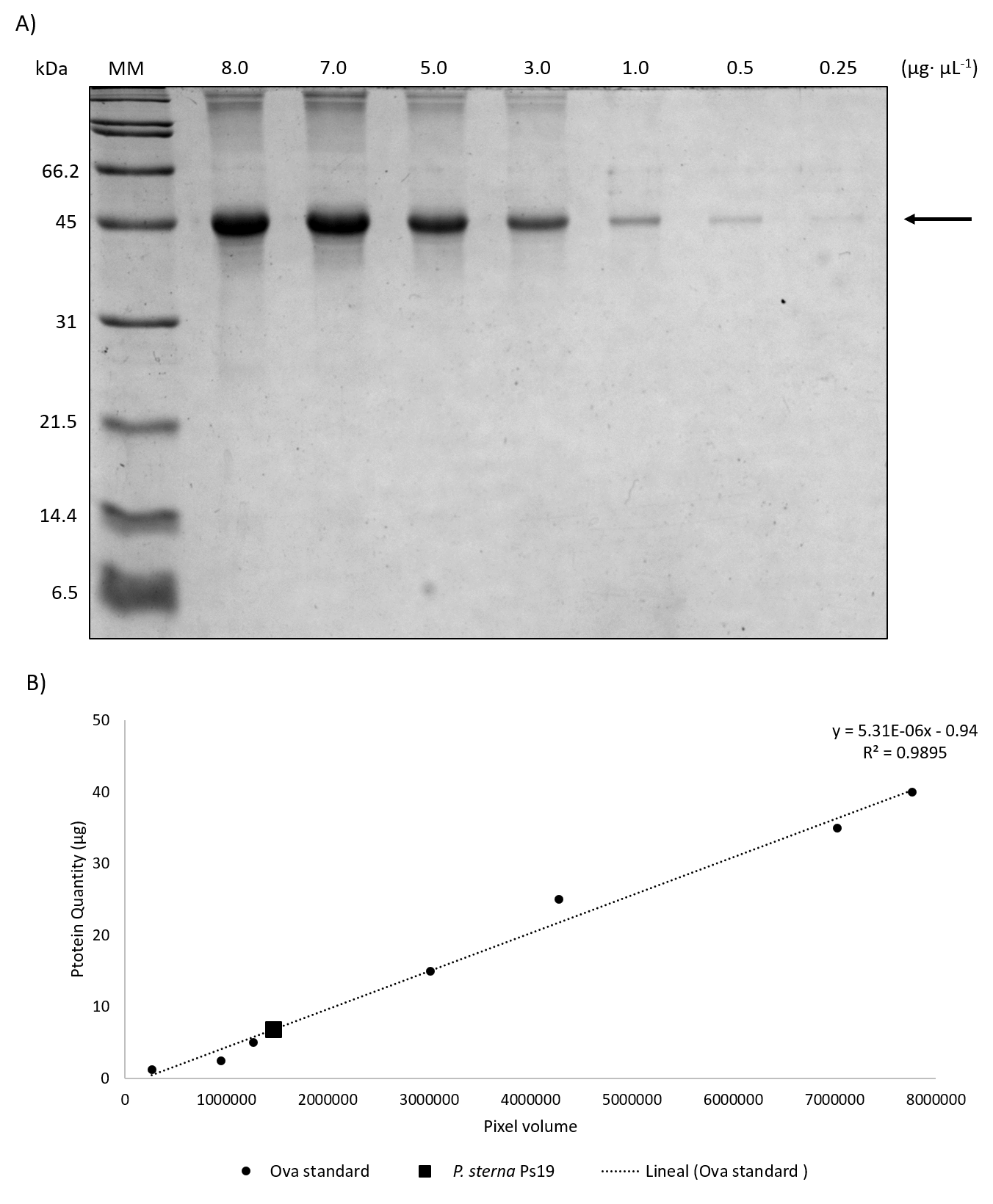

Supplement: S3 Fig — (A) SDS-PAGE 16% polyacrylamide gel. Ovalbumin standard curve indicated by an arrow (8.0–0.25 μg·μL-1) stained with CBB for pixel density determination to calculate the linear equation and quantify protein bands. MM: molecular marker; Ovalbumin concentrations (μg·μL-1). (B) Ovalbumin standard curve graphic. The axes represent pixel volume and protein quantity (μg). Circles represent Ovalbumin (μg), the square corresponds to purified Pteria sterna protein. (TIF) [file pone.0230431.s003.tif]

MM

AIM

X

X

X

ASM

X

X

X

X

66.2

45

31

21.5

14.4

6.5

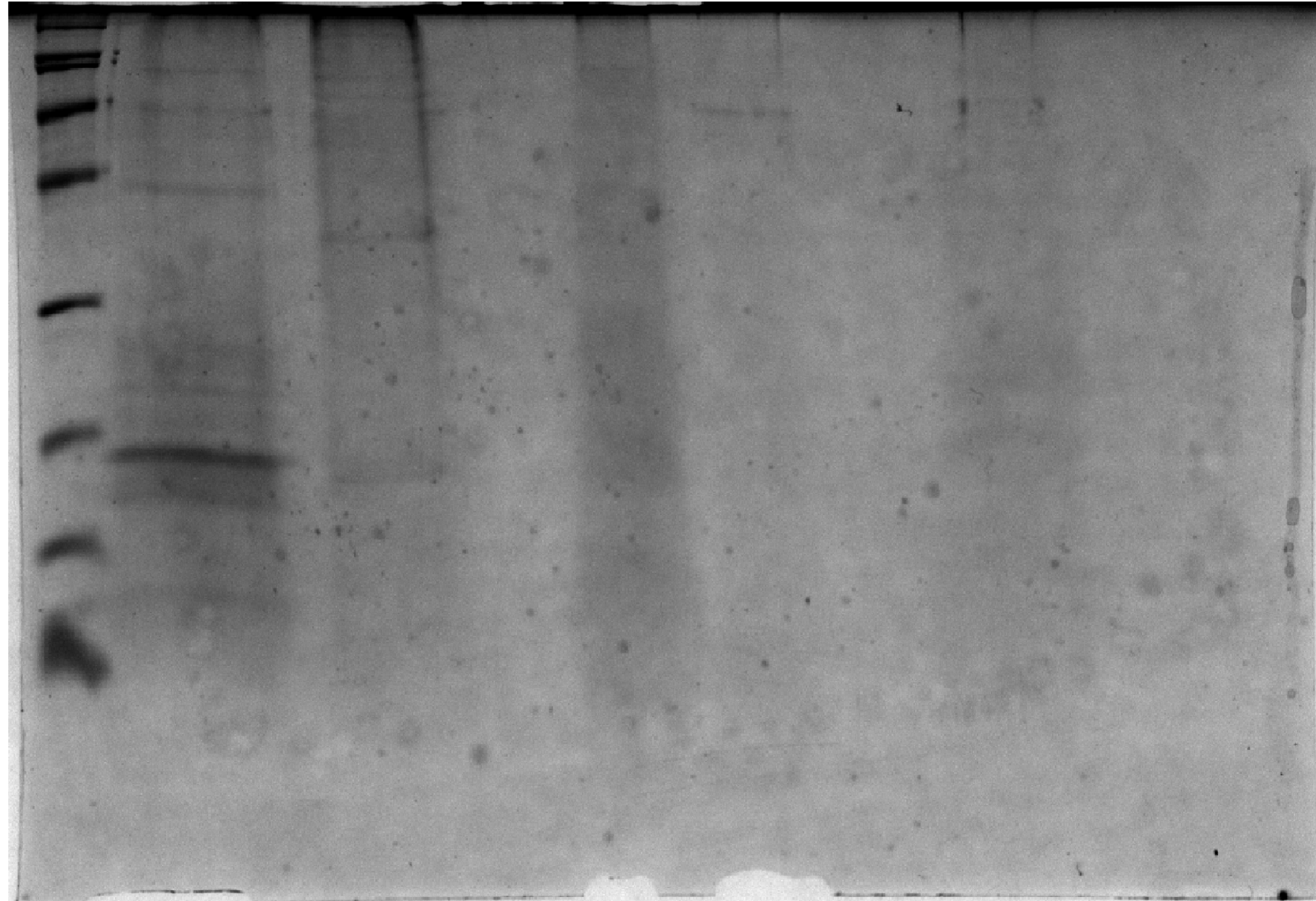

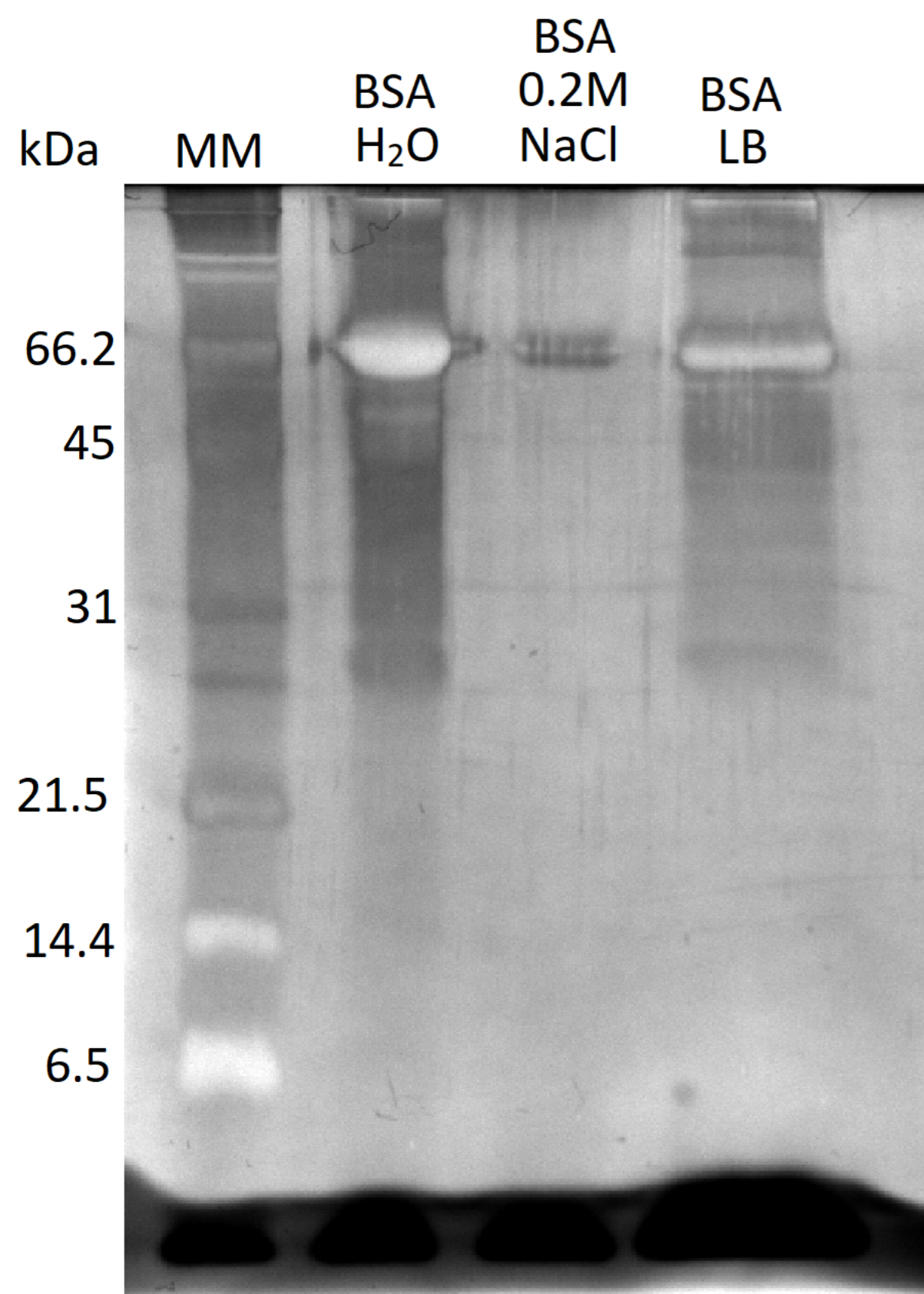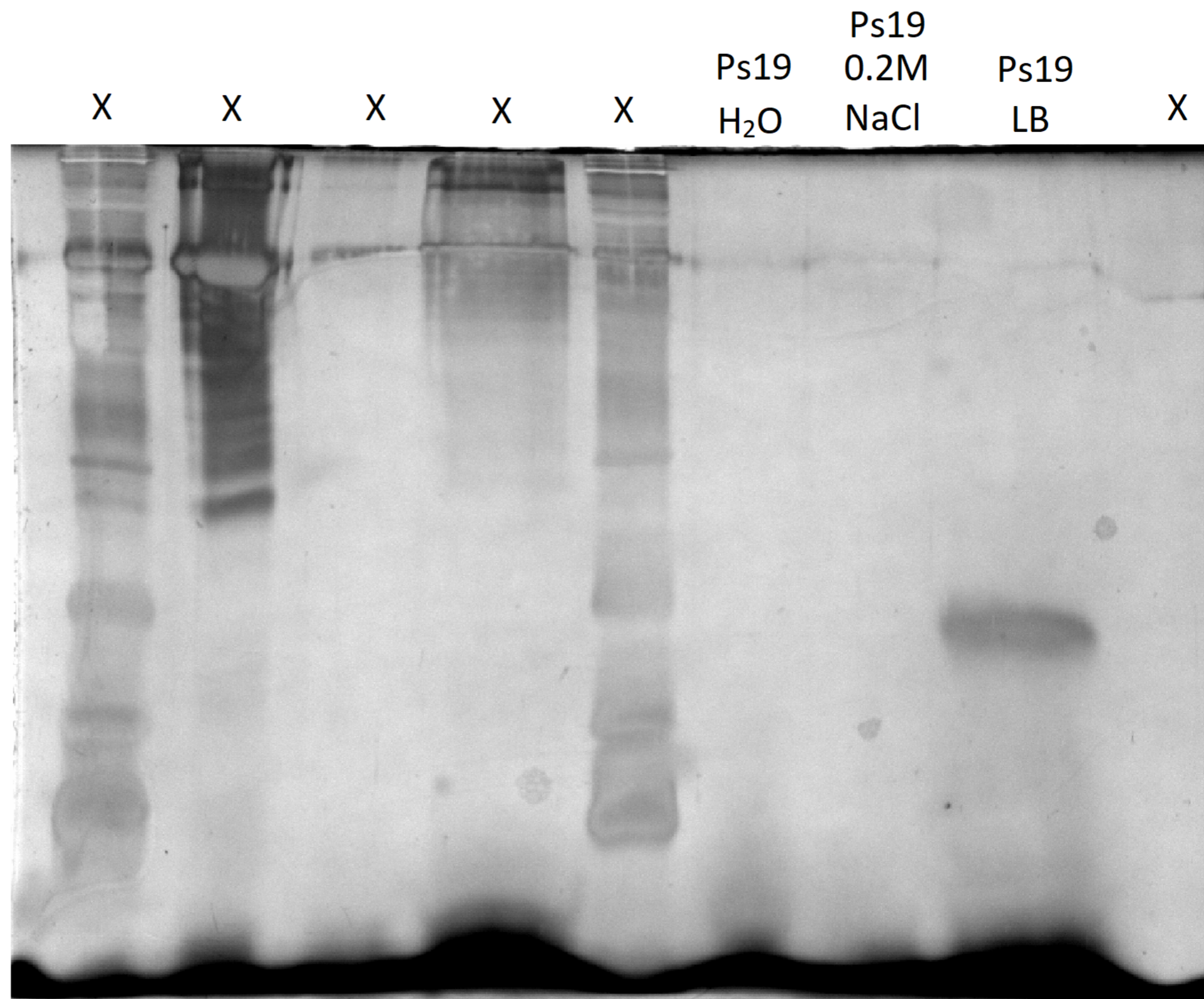

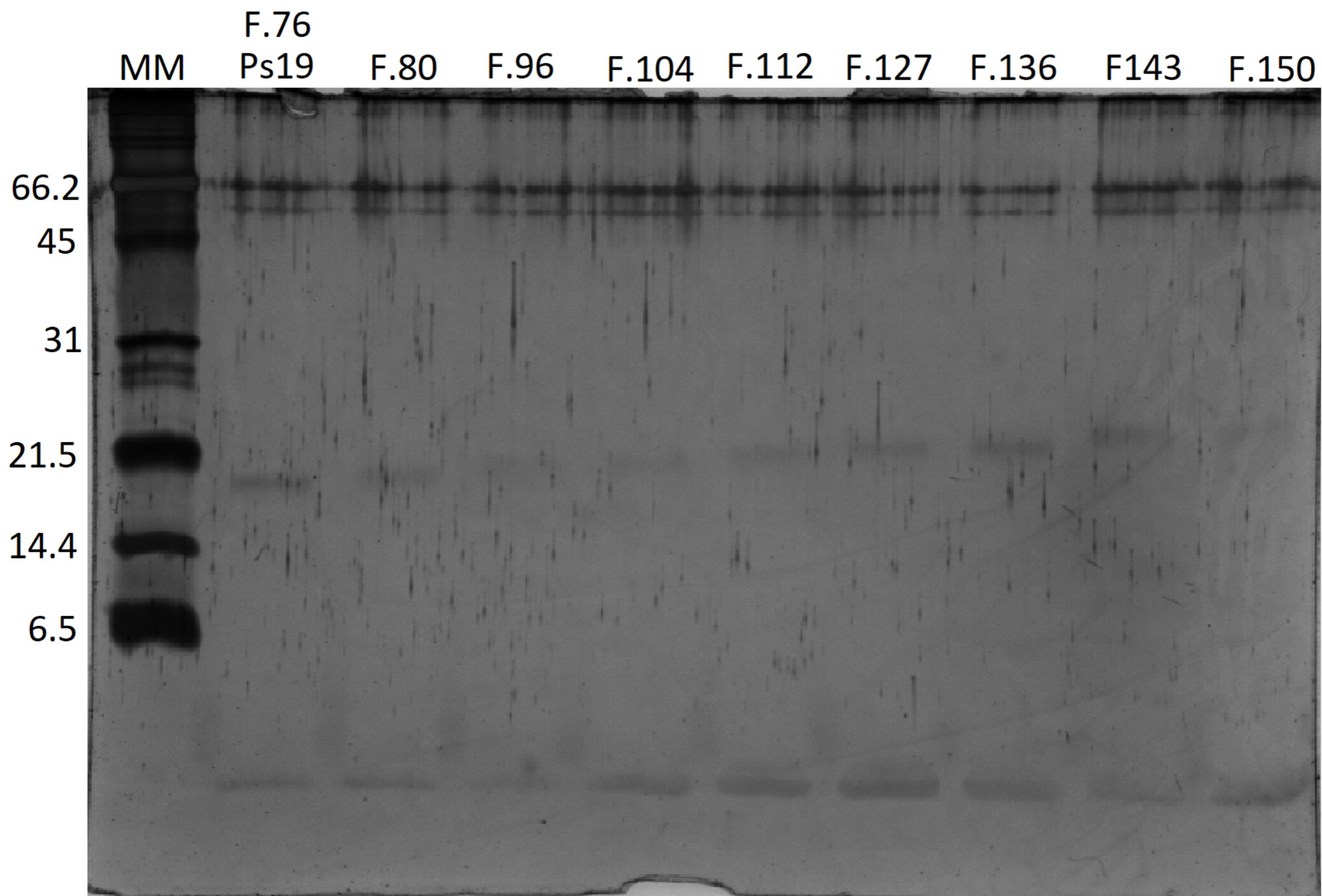

# Ovoalbumin curve

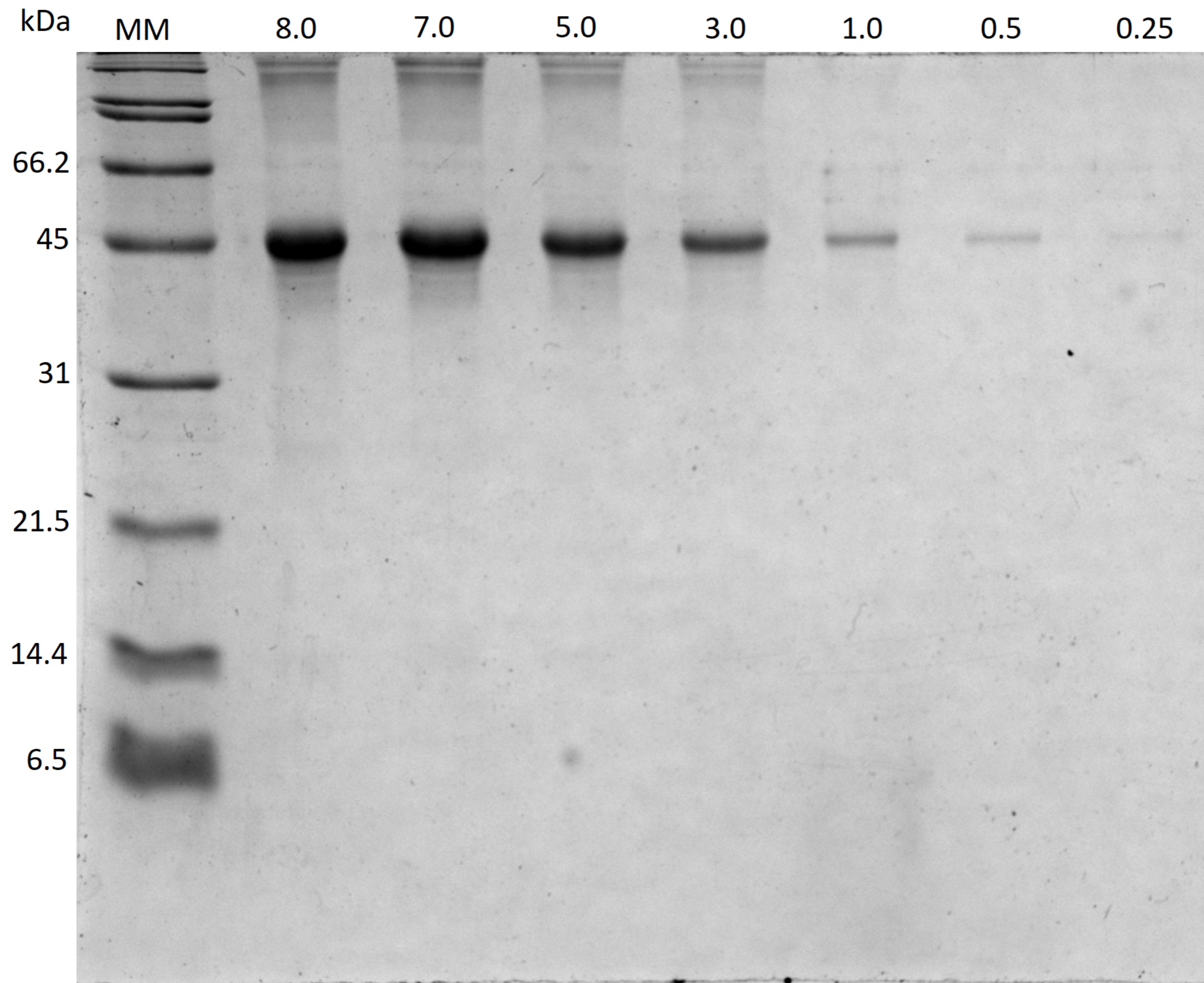

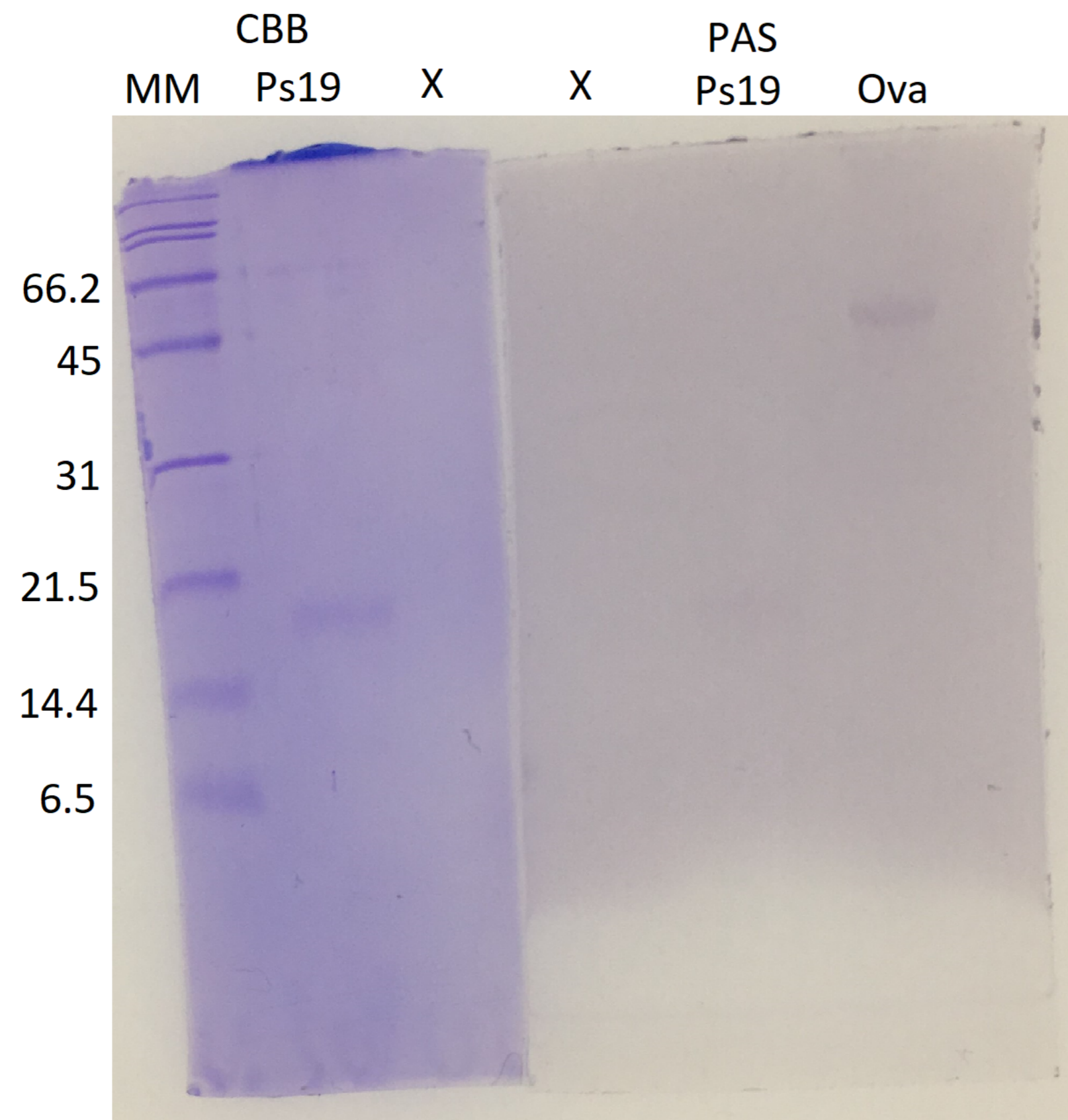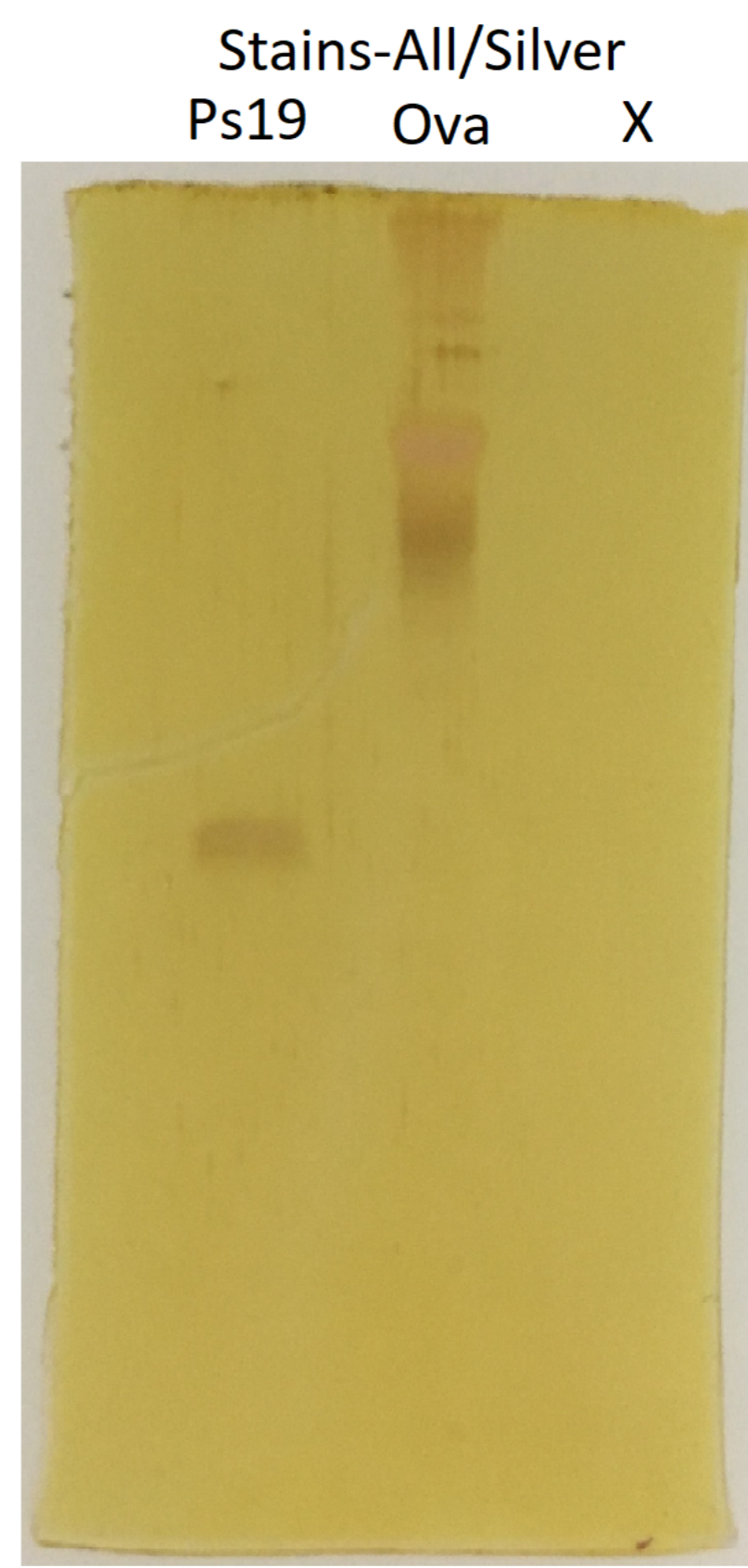

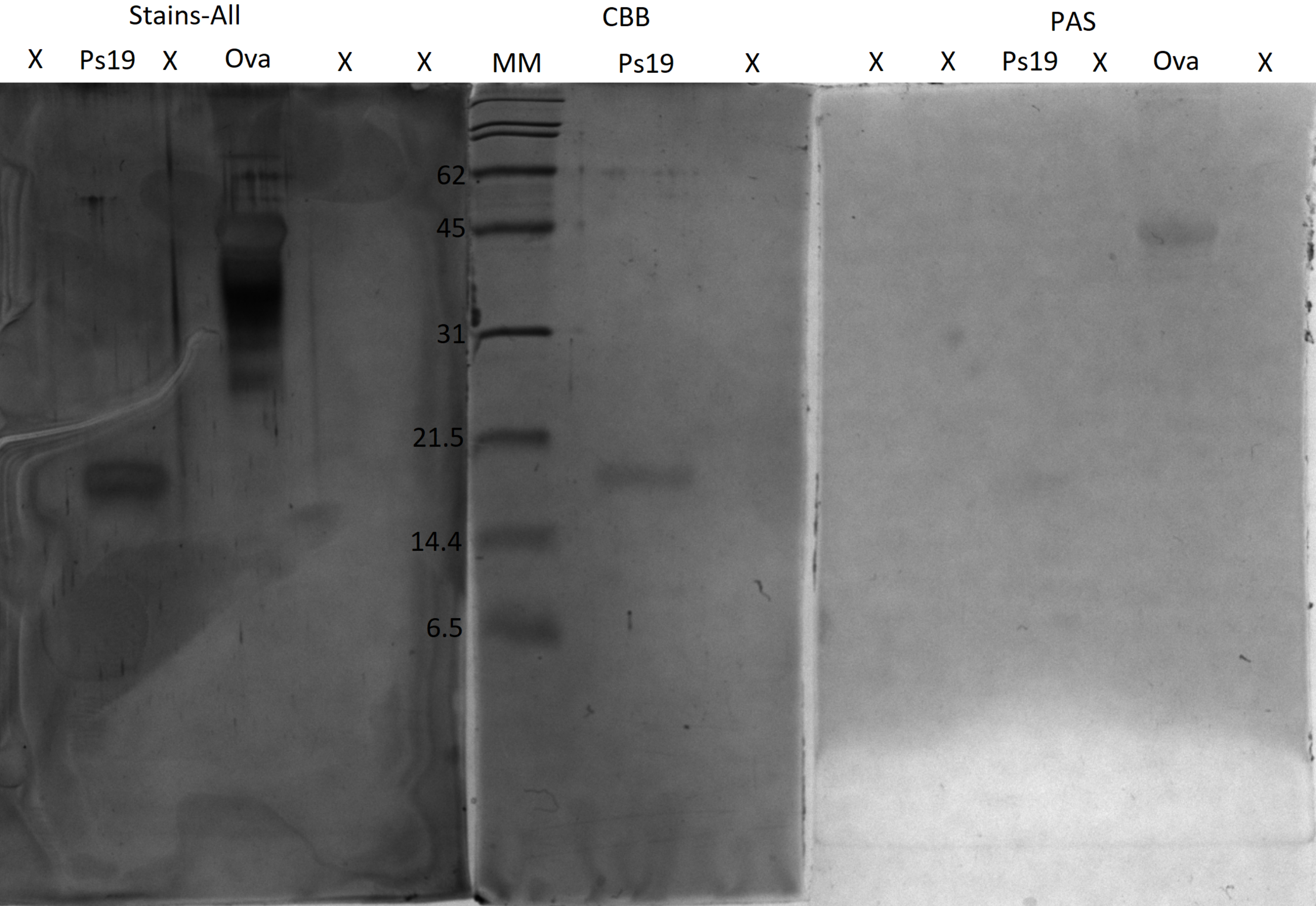

Silver stain

MM

Ps19

X

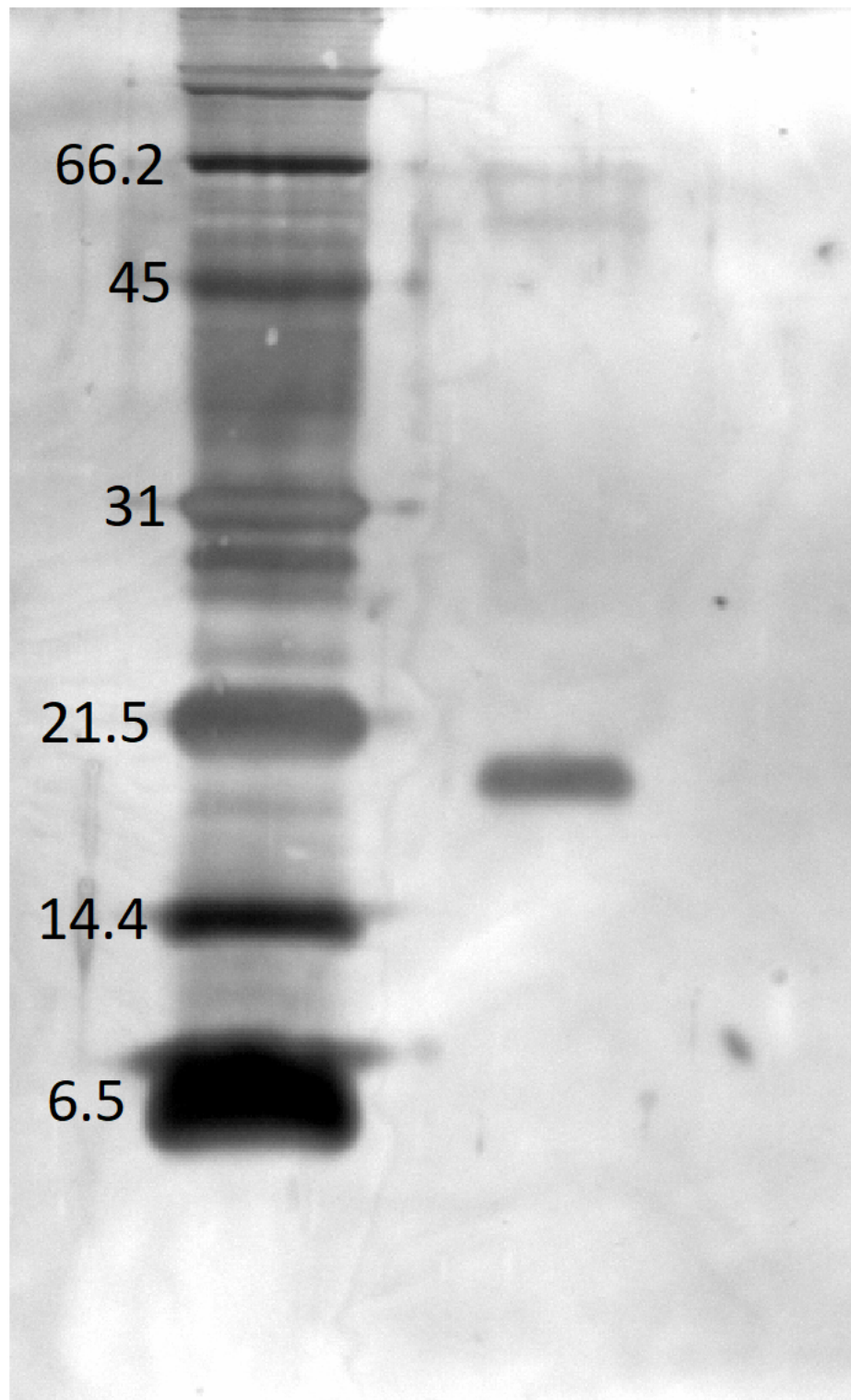

Supplement: S1 Raw image — (PDF) [file pone.0230431.s004.pdf]
